# Rates of interlock screw back-out are high with the retrograde femoral nailing advanced system for distal femur fractures

**DOI:** 10.1007/s00590-024-04006-5

**Published:** 2024-05-28

**Authors:** Rahul Bhale, Sean T. Campbell, Ellen Fitzpatrick, Gillian Soles, Mark Lee, Augustine M. Saiz

**Affiliations:** Davis Department of Orthopaedic Surgery, University of California, 4860 Y St #1700, Sacramento, CA 95817 USA

**Keywords:** Distal femur fracture, Femoral nail, Intramedullary nail, Retrograde femoral nail, Retrograde femoral nailing advanced

## Abstract

**Purpose:**

The retrograde femoral nailing advanced (RFNA) system (DePuy synthes) is a commonly used implant for the fixation of low distal femur and periprosthetic fractures. There is concern that the rate of distal interlock screw back-out may be higher for the RFNA compared to other nails (ON). The purpose of this study was to evaluate the incidence of interlock screw back-out and associated screw removal for RFNA versus ON, along with associated risk factors.

**Methods:**

A retrospective comparative study of patients who underwent retrograde nailing for a distal femur fracture at an academic level one trauma center was performed. The incidence of distal interlock screw back-out and need for screw removal were compared for RFNA versus a propensity score matched cohort who received other nails.

**Results:**

One hundred and ten patients underwent retrograde nailing with the RFNA for a distal femur fracture from 2015 to 2022 (average age: 66, BMI: 32, 52.7% smokers, 54.5% female, 61.8%). There was a significantly higher rate of interlock back-out in the RFNA group compared to the ON (27 patients, 24.5% vs 12 patients, 10.9%, *p* = 0.01), which occurred 6.3 weeks postoperatively. Screw removal rates for back-out were not significantly different for the RFNA group versus ON (8 patients, 7.3% vs 3 patients, 2.7%, *p* = 0.12).

**Conclusion:**

In this retrospective comparative study of distal femur fractures treated with retrograde nailing, the RFNA implant was associated with an increased risk of distal interlock screw back-out compared to other nails.

## Introduction

Distal femur fractures affect 8.7/100,000 patients per year [1, 2]. The average age at the time of injury has been reported to be 62.2 years, though patients typically present in a bimodal distribution of young and elderly patients [1, 3–6]. There has been a recent focus on obtaining fixation stable enough to withstand early weight bearing during the treatment of these injuries in the geriatric population [7]. High complication rates continue to be reported, particularly with lateral plate fixation alone [8–11]. Emerging literature has described retrograde medullary nailing as an effective technique for distal femur fractures, even very low and periprosthetic fractures [12–16].

Modern retrograde nail systems incorporate multiple distal interlocking bolt options to increase fixation in the short segment. The DePuy Synthes (West Chester, PA) Retrograde Femoral Nailing Advanced (RFNA) is a newer implant that has gained recent popularity for the treatment of distal femur fractures [17]. In theory, this implant contains a “locking polymer” within the distal nail body to constrain the distal interlock screws and create a fixed angle relationship between the screws and nail. Anecdotally, the authors have noted a higher-than-expected rate of distal interlock screw back-out with this nail when used to treat distal femur fractures.

The purpose of this study was to evaluate the rate of distal interlock screw back-out for the RFNA versus other nails. Secondary aims included (1) To determine the association of distal interlock screw back-out with nonunion and the need for revision surgeries, including screw removal or revision fixation, (2) To determine if there was a difference in interlock screw back-out rates between periprosthetic and native distal femur fractures, (3) To determine if early weight bearing was predictive of interlock screw back-out, and (4) To determine if geriatric patients (age at least 65 years) were more likely to experience interlock screw back-out. We hypothesized that the rate of distal interlock screw back-out would be higher with the RFNA than other nails, and that back-out would be more likely in geriatric patients, periprosthetic fractures, and patients allowed to weight bear as tolerated immediately postoperatively.

## Materials and methods

After Institutional Review Board (IRB) approval, the Electronic Medical Record (EMR) was queried for patients (age 18+) who underwent retrograde nail fixation for a distal femur fracture (OTA/AO type 33A-C) at a single academic level 1 trauma center from January 2014 to January 2023. Patients were excluded if they were under the age of 18 at the time of surgery, had a pathological fracture, or had combination plate-nail fixation.

Patient characteristics including age, sex, BMI, and smoking status were collected from the EMR. OTA/AO classification and injury characteristics were determined by a review of preoperative injury radiographs and computed tomography (CT) imaging. The implant manufacturer was recorded. Patient follow-up length was recorded. Interlock screw back-out and associated screw removal were assessed regardless of follow-up length; while, nonunion and associated revision fixation were assessed only for patients with at least a three-month follow-up.

The anteroposterior (AP), lateral, and oblique views were evaluated for each patient at their interval post-operative visits, with a step-off relative to the cortex on any view greater than 1 mm change, compared to immediate post-operative radiographs, representing interlock screw back-out. Radiographs and patient charts were evaluated serially to determine the presence of nonunion and the need for revision surgery for screw removal or revision fixation among patients with at least 3 month follow-up. Postoperative weight bearing prescription was recorded.

### Statistical analyses

The cohort of patients who underwent surgery with the RFNA was identified, and a second propensity-score-matched cohort of patients treated with other retrograde nails (ON) was generated for comparison. Propensity score matching was performed using a logistic regression model including covariates such as age, sex, BMI, and smoking status. To confirm, T tests and Chi-squared analyses were used to ensure there were non-significant differences in patient characteristics for the RFNA and other retrograde nail-type cohorts.

Comparative chi-squared analyses were implemented to determine differences in rates of interlock screw back-out, nonunion, and revision rates between the RFNA and other retrograde nail-type cohorts. Sub-analyses using logistic regression were conducted to determine if an immediate weight bearing at tolerated (WBAT) recommendation was predictive of distal interlock screw back-out. We also performed a sub-analysis of the RFNA cohort to compare the rate of interlock screw back-out among geriatric and non-geriatric patients. Power analyses were calculated for two-sample tests for proportions by using the standard normal distribution functions incorporating our study findings and *α* = 0.05. Sufficient power was defined to be at least 0.8. All statistical analyses were performed using the XLMiner Analysis Toolpak (Frontline Systems Inc., Incline Village, NV, USA).

## Results

**Fig. 1:**
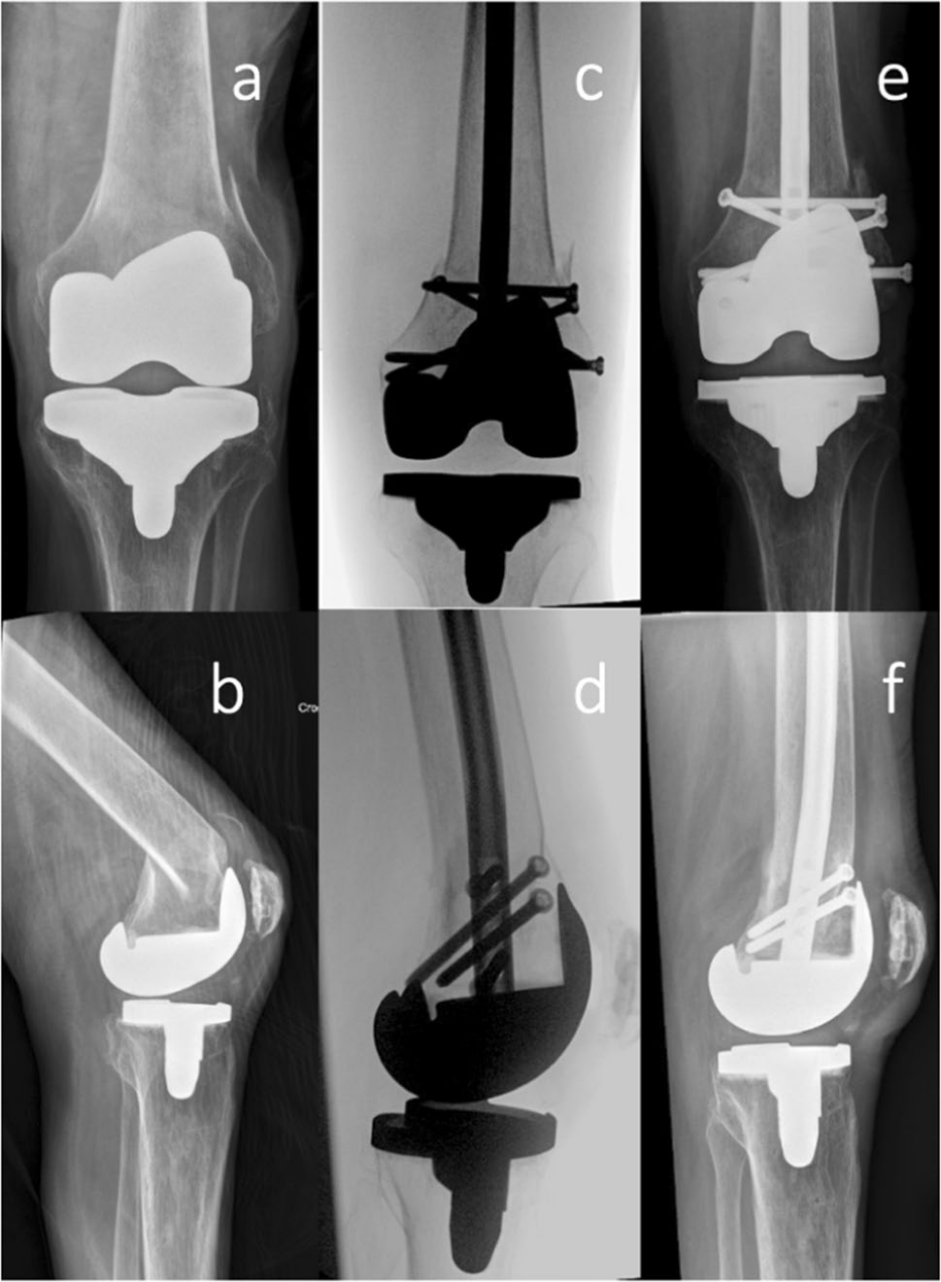
Injury AP (**a**) and lateral (**b**) radiographs demonstrating a low periprosthetic distal femur fracture adjacent to a knee arthroplasty component. Intraoperative AP (**c**) and lateral (**d**) fluoroscopic images following reduction and surgical fixation using the RFNA. Six-week follow-up AP (**e**) and lateral (**f**) radiographs demonstrating unchanged alignment and 3.9 mm distal interlock screw back-out (note the lateral to medial distal-most screw position in image (**e**)

In total, 311 patients met the inclusion criteria. Of those, 110 patients were treated with the RFNA. The matched cohort was treated with the Zimmer Natural Nail System (23 patients, 20.9%) and the DePuy Synthes Retrograde/Antegrade Femoral Nail (RAFN) (87 patients, 79.1%). Patient characteristics of the two cohorts are reported in Table 1. Forty-seven patients (42.7%) in the RFNA cohort had periprosthetic fractures adjacent to a total knee arthroplasty versus 36 patients (32.7%) in the ON cohort, which was not a statistically significant difference (*p* = 0.1). (Fig. 1) The average follow-up length was 21.2 weeks (5.3 months). 136 patients (61.8%) had at least a 3-month follow-up and could be considered for nonunion outcome analysis.

**Table 1:** Patient characteristics for RFNA versus other nail types

	RFNA (*n* = 110)	Other nail types (*n* = 110)	*p*-Value
Age (years)	66	65	0.2
Sex—Female	60 (54.5%)	62 (56.4%)	0.8
BMI	32	33	0.2
Smoking status	58 (52.7%)	54 (49.1%)	0.6

All differences were non-significant (*p*-value > 0.05)

**Fig. 2:**
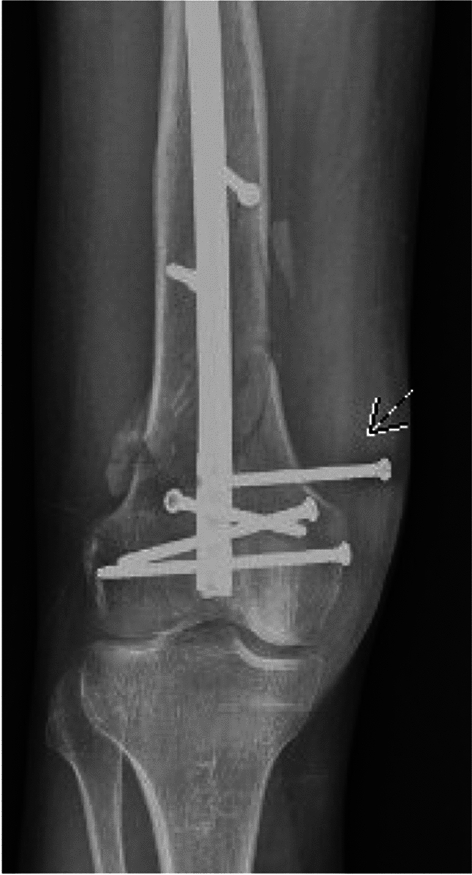
Follow-up AP radiograph demonstrating 27.7 mm distal interlock screw back-out four weeks after treatment with RFNA. The alignment remained unchanged

Thirty-nine total patients (17.7%) had a postoperative course complicated by distal interlock screw back-out. In the RFNA cohort, 27 patients (24.5%) experienced back-out, compared with 12 in the ON cohort (10.9%), which was a statistically significant difference (*p* = 0.01). Overall, the rates of back-out based on screw type were: proximal lateral to medial (19 patients, 48.7%), distal lateral to medial (11 patients, 28.2%), and lateral oblique (9 patients, 23.1%). (Table 2). On average, interlock screw back-out was noted at 6 weeks postoperatively. Eight patients (7.3%) in the RFNA cohort and 3 patients (2.7%) in the ON cohort underwent screw removal for symptomatic back-out, which was not a statistically significant difference (*p* = 0.12). (Fig. 2).

**Table 2:** Rates of interlock screw back-out based on screw type for the RFNA and ON cohorts

	RFNA (*n* = 27)	Other nail types (*n* = 12)	Overall (*n* = 39)
Distal lateral to medial	9 (33.3%)	2 (16.7%)	11 (28.2%)
Proximal lateral to medial	14 (51.9%)	5 (41.7%)	19 (48.7%)
Lateral oblique	4 (14.8%)	5 (41.7%)	9 (23.1%)
Medial oblique	0 (0%)	0 (0%)	0 (0%)

Of the 136 patients with at least a 3-month follow-up length, 62 patients (45.6%) were in the RFNA cohort and 74 patients (54.4%) were in the ON cohort. One patient (1.6%) in the RFNA and one patient (1.4%) in the ON cohort experienced nonunion and underwent a revision fixation. Neither of these patients experienced interlock screw back-out prior to nonunion. The patient in the ON cohort experienced interlock screw breakage; while, the patient in the RFNA cohort developed a septic nonunion, without associated interlock screw back-out.

Of the 27 patients in the RFNA cohort who experienced interlock screw back-out, 11 (40.7%) had periprosthetic fractures and 16 (59.3%) had native distal femur fractures (*p* = 0.2). 15 (55.6%) were recommended to be weight bearing as tolerated (WBAT) immediately, and 12 (44.4%) were recommended to be non-weight bearing (NWB), which was not a statistically significant difference (*p* = 0.4). 21 (77.8%) were geriatric (age over 65 years) versus 6 patients (22.2%) who were under 65, which was a statistically significant difference (*p* < 0.01).

There was sufficient statistical power for the significantly increased rate of interlock screw back-out for RFNA versus ON (0.97) and for geriatric versus younger patients (0.98). However, there was insufficient statistical power for the non-significant differences between screw removal for RFNA versus ON (0.61), periprosthetic versus native distal femur fractures (0.29), and early versus delayed weight bearing (0.13).

## Discussion

In this retrospective chart review study, we sought to determine if patients who underwent distal femur fracture surgery with the Synthes RFNA had a higher rate of screw back-out compared to other retrograde nail types. We found that there was a significantly higher rate of interlock screw back-out for RFNA compared to other retrograde nail types, which occurred at an average of 6.3 weeks postoperatively. These were more commonly the proximal and distal lateral to medial interlock screws, particularly in the RFNA cohort. Geriatric patients were significantly more likely to experience interlock screw back-out. There was no increased rate of unplanned reoperation among patients who experienced back-out, but our study was not powered adequately to fully assess this outcome.

Interlock screws have been associated with pain and reoperation in the literature. Shah et al. [18] retrospectively reviewed 31 patients who received retrograde intramedullary nailing for distal femur fractures, all of whom achieved successful union, though six required symptomatic distal interlock screw removal. Hamaker et al. [19] reported a rate of symptomatic screw removal in 12% of patients and noted that significant risk factors included distal interlocking screws placed within 40 mm of the articular surface [19]. In this study, we found a higher rate of interlock screws back-out with the RFNA implant compared to other nails. While we did not identify an increased rate of removal, this was not the primary outcome of the study, and the study is likely underpowered to detect differences. We are also limited by a lower follow-up duration. Despite this, we believe that any instance of back-out is undesirable, as it may become symptomatic as swelling subsides and over time. Additionally, the degree of symptoms may not warrant removal, but still be bothersome to the patient, which is more difficult to measure. Even one reoperation in a frail geriatric patient should be avoided if possible.

In a similar investigation, Minhas et al. recently conducted a case series analysis of patients who experienced distal interlock screw back-out after undergoing distal femur fracture fixation with the RFNA nail [20]. It was reported that of the 27 patients who underwent fixation, 8 patients experienced distal interlock screw back-out at an average of 61 days postoperatively. All of these patients complained of implant prominence and pain along the medial or lateral aspect of the knee, five of whom underwent screw removal. The oblique distal interlock screws comprised 62% of screw back-outs. Associated patient discomfort, need for reoperation, and healthcare costs warrant implant re-design.

This study has a number of limitations. These include the retrospective nature, with the possibility of patient selection bias. Additionally, although a large cohort compared to prior literature, this study was at risk of being underpowered to detect a difference. Specifically, due to limited follow-up and statistical power, we were unable to adequately assess whether interlock back-out was associated with nonunion and reoperation. Furthermore, we could not assess if fracture patterns, such as periprosthetic versus native distal femur fractures or postoperative weight bearing protocols were predictive of interlock screw back-out. Also, the patients evaluated in this study all received surgical treatment at a single institution, which may lead to challenges with generalizability due to differing patient populations and surgeon experiences. Further, this study looked at retrograde IMN fixation in isolation for distal femur fracture. The effect of supplemental plating remains unknown. Therefore, future studies are warranted to evaluate other indicators or risk factors for complications including nonunion following retrograde intramedullary nail fixation. Another limitation of this study is the lack of patient-reported outcome measures (PROMs) in our retrospective cohort, which would have provided valuable information regarding patients’ pain and functional status. Future studies should seek correlations between interlock screw back-out and these outcome measures to determine clinical relevance.

The Synthes Retrograde Femoral Nail Advanced implant had a higher rate of interlock screw back-out when used for the treatment of distal femur fractures compared to other nails in a matched patient cohort. However, interlock screw back-out was not associated with immediate weight bearing nor fracture nonunion. Geriatric patients were more likely to experience interlock screw back-out. This information may be of use to surgeons who treat these injuries and may assist with implant selection and counseling regarding post-operative sequelae.
